# In Your Face: Startle to Emotional Facial Expressions Depends on Face Direction

**DOI:** 10.1177/2041669517694396

**Published:** 2017-02-01

**Authors:** Ole Åsli, Henriette Michalsen, Morten Øvervoll

**Affiliations:** Department of Psychology, University of Tromsø—The Arctic University of Norway, Tromsø, Norway

**Keywords:** startle, facial expressions, emotion

## Abstract

Although faces are often included in the broad category of emotional visual stimuli, the affective impact of different facial expressions is not well documented. The present experiment investigated startle electromyographic responses to pictures of neutral, happy, angry, and fearful facial expressions, with a frontal face direction (directed) and at a 45° angle to the left (averted). Results showed that emotional facial expressions interact with face direction to produce startle potentiation: Greater responses were found for angry expressions, compared with fear and neutrality, with directed faces. When faces were averted, fear and neutrality produced larger responses compared with anger and happiness. These results are in line with the notion that startle is potentiated to stimuli signaling threat. That is, a forward directed angry face may signal a threat toward the observer, and a fearful face directed to the side may signal a possible threat in the environment.

Emotional visual stimuli seem to receive a prioritized processing in relation to nonemotional ones (*The Cambridge Handbook of Human Affective Neuroscience*, 2012). As such, these affective stimuli have been widely used to elicit emotions in research, and most used are pictures of emotional scenes. Negative emotional scenes typically produce more intense reactions compared with neutral and positive scenes (Cuthbert, Bradley, & Lang, 1996; Lang, Bradley, & Cuthbert, 1998; Vrana, Spence, & Lang, 1988). Furthermore, pictures containing emotional faces have often been included in the broader category of emotional scenes (Lang & Greenwald, 1988). However, the effects of emotional facial expressions are not as distinct as the effects of emotional scenes (Alpers, Adolph, & Pauli, 2011; Paulus, Musial, & Renn, 2014). In addition, pictures of faces may vary in relevance to the observer based on head direction. That is, an emotional face directed at you is arguably more relevant than one turned in another direction. In the present study, the effects of different emotional facial expressions as well as the effect of face direction was investigated.

In contrast to the extensive use of emotional scenes in psychophysiological research, there are few published studies utilizing exclusively emotional faces as stimuli. In addition, the limited research on the topic has revealed inconsistent results employing both electrodermal responses (Dimberg, 1982; Merckelbach, van Hout, van den Hout, & Mersch, 1989; Vrana & Gross, 2004) and heart rate (Dimberg, 1982; Johnsen, Thayer, & Hugdahl, 1995; Vrana & Gross, 2004). Utilizing electrodermal responses, some studies have found similar reactions to angry and happy facial expressions (Dimberg, 1982; Dimberg & Petterson, 2000; Vrana & Gross, 2004). Considering heart rate, two studies have found the expected deceleration to angry facial expressions and acceleration to happy expressions (Johnsen et al., 1995; Vrana & Gross, 2004)*.* However, Vrana and Gross (2004) found deceleration also for neutral faces, and Hess, Philippot, and Blairy (1998) did not find any differences between angry and happy expressions.

In the present study, the startle reflex was used as an index of emotional activation. The startle reflex is a simple defensive response to sudden and intense stimuli (e.g., a loud noise) that in humans involves a rapid closure of the eyelids. It has been well demonstrated that the amplitude of the startle eye blink varies with the emotional content of the background stimulus viewed simultaneously (Lang, Bradley, & Cuthbert, 1990), and it seems to be produced by the priming of the startle circuitry by the amygdala (Davis, 1992). Similar, Angrilli et al. (1996) found that the right amygdala was vital for emotional modulation of the startle response. The typical emotional modulation is increased startle to negative pictures and, to some lesser extent, inhibited startle to positive pictures (Cuthbert et al., 1996; Lang et al., 1998; Vrana et al., 1988). As such, there is a close relation between startle potentiation and valence, and in addition, a relation between the amount of potentiation and arousal for negative valence pictures (Bradley, Codispoti, & Lang, 2006). However, research on startle in relation to emotional facial expressions has revealed a less clear pattern (Alpers et al., 2011; Paulus et al., 2014). Anger seems to consistently potentiate startle, but other than that, the question about which facial stimuli potentiates startle is still unsettled.

Some studies have investigated questions related to startle responding to facial stimuli. Among these, Balaban (1995) found potentiated startle to angry faces and inhibited startle to happy faces in human infants, whereas Waters, Neumann, Henry, Craske, and Ornitz (2008) did not find modulated startle to neutral and angry facial expressions in four- to eight-year-old children. Anokhin and Golosheykin (2010) found startle potentiation to negative facial expressions (i.e., angry and fearful), with a stronger effect in females. Hess, Sabourin, and Kleck (2007) and Paulus et al. (2014) found startle potentiation to angry emotional faces, but only when the expressers were males. Springer, Rosas, McGetrick, and Bowers (2007), on the other hand, found potentiated startle to angry faces regardless of the sex of the expresser. Furthermore, Alpers et al. (2011) found increased startle to angry facial expressions compared with neutral, but not compared with happy expressions. Dunning, Auriemmo, Castille, and Hajcak (2010) morphed pictures of faces and found potentiated startle to angry faces compared with neutral ones, but only for maximally angry expressions. Even fewer studies have investigated the psychophysiological effect of fearful facial expressions and the differences between subtypes of negative expressions. Nonetheless, Grillon and Charney (2011) found enhanced startle responses to fearful faces but only in a threat situation. Springer et al. (2007) compared the effect of angry and fearful faces and found potentiated startle to angry faces, but not to fearful ones.

The only startle study we are familiar with investigating the effect of gaze direction found greater responding to pictures where the model gazed directly at the observer (Lass-Hennemann et al., 2009). However, this study investigated the effect of pictures of nude females on the startle reaction in men, thereby investigating the effect of a distinct subtype of pleasant stimuli not directly comparable to the stimuli utilized in the present study. Adams and Kleck (2003) investigated behavioral data and argued that facial displays of emotions and gaze direction combines to signal approach or avoidance. They found that anger and happy expressions were more quickly recognized in combination with a direct gaze, and that fear and sad expressions were more quickly decoded with an averted gaze. Later, Adams, and Kleck (2005) found support for a notion that direct gaze would enhance the perception of approach-oriented emotions, and averted eye gaze would enhance the perception of avoidance-oriented emotions. Following this logic, one could expect larger startle responses to anger and happy facial expressions with a frontal directed face, and larger startle to fearful faces with averted facial direction.

In an exploratory part of the experiment, we investigated the startle response to facial stimuli at early lead intervals. A few studies have investigated early lead interval responses (50–500 ms) to pictures of emotional scenes (Bradley et al., 2006; Bradley, Cuthbert, & Lang, 1993; Duval, Lovelace, Aarant, & Filion, 2013). These studies found smaller startle response to more emotionally arousing stimuli, compared with neutral stimuli. This effect, which is opposite to the larger startle response to emotionally arousing stimuli at late intervals, is probably caused by more prepulse inhibition (PPI) to the emotional stimuli. PPI is the effect of which a nonstartling stimulus precedes the startle probe by a short duration and reduces the startle response (Graham, 1975) and is an index of protective processing of the prepulse. Thus, in the present study, the pictures of emotional faces would serve as the prepulse, and an inhibition of the startle response would be an index of the processing of these pictures. Bradley et al. (2006) suggested that affectively engaging stimuli produces more inhibition as they are more demanding to the attentional system. Bradley et al. (1993) argued that more interesting and complex pictures leads to longer periods of “processing protection” compared with simpler stimuli. Thus, producing more PPI and, as such, less startle. Duval et al. (2013) investigated the startle response to pictures of happy, neutral, and angry faces at 300, 800, and 3500 ms. At the early 300 ms lead interval, they found smaller startle response to angry compared with neutral faces, but only when the expresser were males. For female expressers, the results where opposite, with smaller responses to neutral faces compared with angry.

In the present study, we investigated the startle reflex during presentations of pictures displaying angry, fearful, happy, and neutral facial expressions. Faces were directed (toward the viewer) and averted (away from the viewer), and startle eliciting stimuli were presented at an early lead interval (250 ms) and at a late lead interval (3500 ms). Based on the startle literature reviewed earlier, we hypothesized that startle would be potentiated to angry faces compared with neutral and fearful at a late lead interval when faces were directed forward. In addition, based on the work of Adams and Kleck (2003, 2005), we expected increased startle to forward directed approach motivated emotions (anger and happiness) and increased startle to averted directed avoidance motivated emotions (fear). In addition, at the early lead interval, we expected less startle to emotionally arousing stimuli. However, this part of the experiment was more exploratory as the previous research on this effect is scarce. In addition to the startle test, participants evaluated the pictures on scales for valence, arousal, and domination.

## Method

### Participants

Thirty people (13 men, 17 women, age range 17–40, mean age 22.0 years) participated in the study. All participants reported good health and did not report any hearing problems, previous serious disease, or injury. The participants were instructed not to drink caffeinated beverages or use nicotine-containing substances for 3 hr prior to the experiment. They were also told that they could withdraw from the study at any time without giving any reason. Written informed consent was obtained from all participants, who were given two lottery tickets (equivalent to 50 NOK) for their participation or course credit for an introductory psychology class.

### Apparatus and stimuli

The experiment took place in an electrically and acoustically shielded chamber where the temperature was kept at about 20℃. A Bruel and Kjær 2235 Sound Level Precision Meter was used to measure the intensity of auditory stimuli. Programs for experimental control were written by the first author in Coulbourn Human Startle System HSW v. 7.500 – 00 and run on a Microsoft Windows XP based Dell PC that controlled presentation of experimental stimuli and data acquisition.

**Figure 1.**
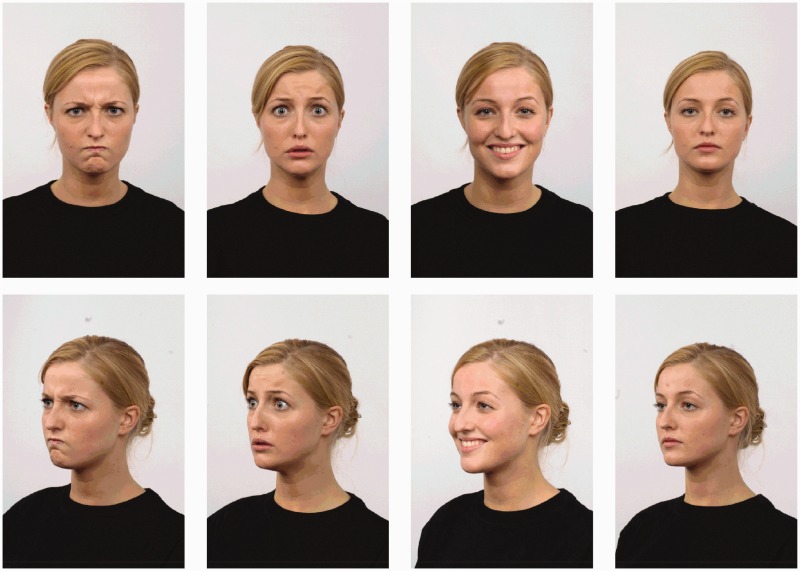
One of the models used in the experiment shows the four facial expressions (left to right: angry, fearful, happy, and neutral) with directed and averted face direction.

Pictures of faces were selected from the Radboud Faces Database (Langner et al., 2010) showing four emotions: angry, fearful, happy, and neutral. Pictures of facial expressions were shown with the expresser facing the observer (i.e., directed) and at a 45° angle to the left (averted) (Figure 1). Four male (model no.: 21, 23, 28, and 71) and four female (model no.: 1, 4, 57, and 58) expressers showed each facial expression, and three male and three female expressers were randomly chosen to be presented to each participant, giving a total of six pictures of each facial expression-head direction combination. Pictures were presented for 5 s and in a random order. Intertrial interval was between 17 and 23 s (mean 20 s). Pictures were presented on a 17-in. monitor situated 70 cm in front of the participants.

Startle-eliciting noise had an intensity of 95 dB (SPL), instantaneous rise time, and duration of 50 ms. The stimuli were delivered through Sennheiser HD 250 headphones. The startle-eliciting noise was presented 250 or 3500 ms after picture onset. Startle-eliciting stimulus was presented at every trial (once per picture).

Startle eyeblink electromyographic responses were recorded from the right orbicularis oculi with two sintered-pellet silver chloride AgCl miniature electrodes (4 mm diameter) filled with Microlyte electrolyte gel (Coulbourn Instruments)*.* Inter-electrode distance was 1.5 to 2 cm. The ground electrode was placed centrally on the forehead. The electromyography (EMG) signal was amplified by a factor of 50,000 and filtered (passing 8–1000 Hz) by a Coulbourn V75-04 bioamplifier*.* The signal was rectified and integrated with a Coulbourn V76-24 contour-following integrator with a 10-ms time constant, and the output was sent to the PC via a LabLinc V interface. A 12 bits A/D board was used. Sampling on each trial began 100 ms prior to onset of the startle stimulus and continued for 200 ms after onset of the stimulus. The sampling rate was 1000 Hz.

Participants rated the pictures for valence, arousal, and domination in the room adjacent to the experimental chamber. Participants used the mouse pointer to indicate the level of valence, arousal, and domination elicited by each picture using a visual analog scale (VAS) on a computer screen. Each participant viewed each picture for as long as they wanted before rating. The instruction read: “Please mark on the line below how you would rate the picture”; for valence, the endpoints were labeled “unpleasant” and “pleasant”; for arousal, the endpoints were labeled “relaxed” and “agitated”; and for domination, the endpoints were labeled “submissive” and “dominant.” The response range for both was from 0 to 100 mm. The program for the VAS was written in, and controlled by, MATLAB version 8.3 with Psychophysics toolbox (Brainard, 1997).

### Procedure

After arrival at the laboratory, the participants sat down in a desk chair, read, and signed the Informed Consent Form. Thereafter, the participants were lead into the experimental chamber and seated in a reclining chair. The subjects were informed of the general purpose of the study and about the stimuli and procedure. They were also told that they could withdraw from the study without giving any reason at any time. The skin below the participants’ right eye was cleaned with a swab containing alcohol and pumice, and the electrodes for measurement of the startle blink EMG were attached. The headphones were attached and the experimental procedure was initiated as described in the Apparatus and Stimuli section. The door to the experimental chamber was closed during all stimulus presentation.

After the startle session, the participants rated the pictures (valence, arousal, and domination) on a computer in the room adjacent to the experimental chamber. The participant viewed each picture for as long as they wanted before rating (more details in the Apparatus and Stimuli section). After the subjective test, the experiment was over, and the participants received the lottery tickets and left.

### Response scoring and data reduction

Startle blink reflexes were scored as the difference between the maximum amplitude of the EMG response in the window from 0–200 ms after noise onset, compared with the mean EMG level for the last 100 ms prior to onset of the startle eliciting noise on that trial. Startle amplitude values were T-transformed (Z-scores multiplied by 10 and add 50). Startle baseline on each trial was calculated as the mean EMG activity in the last 100 ms prior to the startle eliciting stimulus. The average for each trial includes values of zeroes for nonresponse trials. Less than 1% of startle responses was scored as nonresponses.

### Design and analysis

The design was a 2 Face Direction (directed, averted) by 4 Emotion (angry, fearful, happy, neutral) by 2 Lead intervals (250, 3500 ms) within-subject design. Results were considered significant if *p* < .05. Significant main effects or interactions related to the hypothesis were followed-up by contrast analyses. Other main effects or interactions were followed-up by Tukey’s honest significant difference test.

## Results

### Self-reported ratings

#### Valence

In the 2-Face Direction by 4-Emotion within-subject analysis, the main effect of Face Direction was not significant, although there was a weak trend, *F*(1, 29) = 3.03, *p* = .092, η^2 ^= .09, toward more positive ratings for frontal face direction. The main effect of Emotion was significant, *F*(3, 87) = 121.10, *p* < .001, η^2 ^= .81. Contrast analyses revealed the following significant hierarchy from most positive to least positive: Happy > Neutral, *F*(1, 29) = 274.48, *p* < .001, η^2 ^= .90, Neutral > Fearful, *F*(1, 29) = 19.29, *p* < .001, η^2 ^= .40, Fearful > Angry, *F*(1, 29) = 12.03, *p* = .002, η^2 ^= .29. The interaction of Face Direction by Emotion was not significant, *F*(3, 87) = 1.49, *p* < .22, η^2 ^= .05.

#### Arousal

In the 2-Face Direction by 4-Emotion within-subject analysis, the main effect of Face Direction was not significant (*F* < 1). The main effect of Emotion was significant, *F*(3, 87) = 30.10, *p* < .001. Contrast analyses revealed the following significant hierarchy from most arousal to least arousal: Fearful = Angry, Angry > Neutral, *F*(1, 29) = 69.59, *p* < .001, η^2 ^= .71, Neutral = Happy. The interaction of Face Direction by Emotion was not significant (*F* < 1).

#### Dominance

In the 2-Face Direction by 4-Emotion within-subject analysis, the main effect of Face Direction was not significant (*F* < 1). The main effect of Emotion showed a trend, *F*(3, 87) = 2.64, *p* = .054, η^2 ^= .08. The interaction of Face Direction by Emotion was not significant (*F* < 1).

### Startle

**Figure 2.**
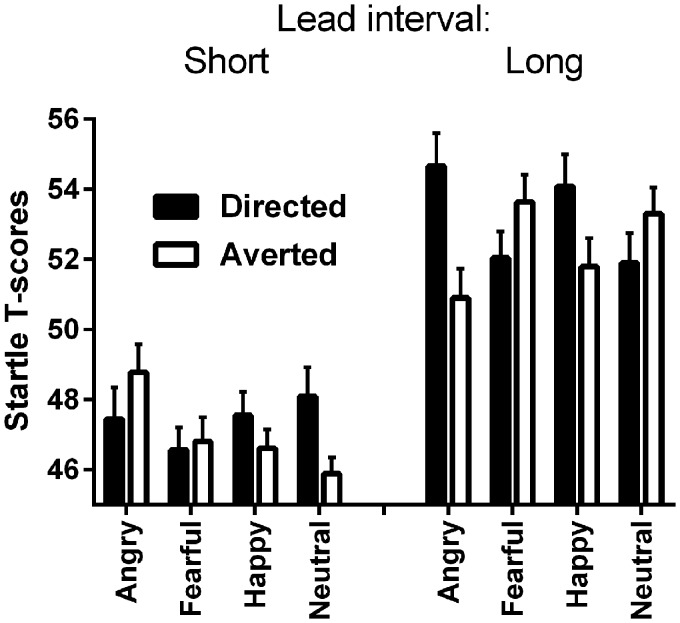
Startle to pictures of difference facial expressions with directed and averted face direction, at short and long lead intervals. Error bars represent +1 standard error of the mean.

In the 2-Face Direction by 4-Emotion by 2-Lead intervals within-subject analysis, there was a significant main effect of Lead interval, *F*(1, 29) = 36.75, *p* < .001, η^2 ^= .56, with greater startle to long lead interval compared with short lead interval. The other main effects were not significant: Emotion: (*F* < 1), Face Direction: *F*(1, 29) = 2.20, *p* = .15, η^2 ^= .07. The interaction of Face Direction by Emotion was significant, *F*(3, 87) = 3.86, *p* = .01, η^2 ^= .12. In addition, the three-way interaction of Face Direction by Emotion by Lead interval was significant, *F*(3, 87) = 8.74, *p* < .001, η^2 ^= .23 (Figure 2).

#### Long Lead Interval

Following up the 3-way interaction, contrast analysis revealed the following differences for the long lead interval condition: Planned comparisons showed greater startle to angry facial expressions with frontal directed face compared with the same expression where the face was directed to the side, *F*(1, 29) = 10.75, *p* = .003, η^2 ^= .27. The same difference was found for happy faces, *F*(1, 29) = 4.65, *p* = .04, η^2 ^= .14. For fearful faces, the difference between face direction was not significant, but there was a trend toward greater startle when the faces were directed to the side, *F*(1, 29) = 3.84, *p* = .06, η^2 ^= .12. There was no difference between face directions for neutral expressions (*F* < 2).

Planned comparison contrast analysis showed greater startle to angry facial expressions with frontal face direction compared with fearful, *F*(1, 29) = 6.07, *p* = .020, η^2 ^= .17, and neutral, *F*(1, 29) = 4.56, *p* = .041, η^2 ^= .14, facial expressions with the same face direction. Fearful facial expressions with left face direction elicited greater startle than happy, *F*(1, 29) = 4.51, *p* = .042, η^2 ^= .13, and angry, *F*(1, 29) = 10.19, *p* = .003, η^2 ^= .26, facial expressions with the same face direction. In addition, neutral facial expression with left face direction elicited greater startle than angry with the same direction, *F*(1, 29) = 6.23, *p* = .019, η^2 ^= .18.

#### Short Lead Interval

Follow-up analysis revealed fewer differences for the short lead interval condition: Planned comparison contrast analysis showed greater startle to neutral facial expression when the face was directed forward compared with the same facial expression when the face was directed to the side, *F*(1, 29) = 7.34, *p* = .011, η^2 ^= .20. In addition, neutral facial expression elicited greater startle than fearful facial expressions when faces were directed forward, *F*(1, 29) = 6.05, *p* = .020, η^2 ^= .17. With face direction to the side angry facial expression produced larger startle than fearful, *F*(1, 29) = 5.89, *p* = .022, η^2 ^= .17, happy, *F*(1, 29) = 8.83, *p* = .006, η^2 ^= .23, and neutral, *F*(1, 29) = 11.45, *p* = .002, η^2 ^= .28, expressions.

## Discussion

In the present study, we compared the startle response to pictures of faces with a forward direction (directed) with faces directed to the side (averted). Our analyses of the long lead interval condition revealed that directed angry faces elicited greater startle than averted angry faces. Larger startle to directed faces was also evident for happy faces. For the fearful faces, there was a trend toward greater startle to the averted compared with the ones directed forward. As far as we know, this is the first study comparing startle reactions to faces directed to the side to the standard pictures of faces directed forward. These results fit well with the findings from Adams and Kleck (2005) that direct gaze enhances the perception of approach-oriented emotions (anger and happiness), and averted eye gaze enhances the perception of avoidance-oriented emotions (fear).

These results could also be in line with findings from the imaging literature: N’Diaye et al. (2009) and Hadjikhani et al. (2008) found stronger amygdala activation to fearful facial expressions with an averted gaze, and N’Diaye et al. (2009) found stronger amygdala activation angry facial expressions with a directed gaze. N’Diaye et al. (2009) argued that amygdala activation follows a self-relevance model, where a forward directed angry face may signal a threat toward the observer, and where a fearful face directed to the side may signal a possible threat in the environment.

Happy faces are not expected to signal threat. However, a happy face directed at you is more relevant to you than one directed to the side. As such, it could be that startle responding to facial stimuli mimics the activation of the amygdala and is in line with the “relevance detector model” (Sander et al., 2003), which postulates a general role for the amygdala in recognizing biologically and socially important information. In addition, Alpers et al. (2011) argued that a facial expression of happiness, without the social context, may not yield enough information about the situation to prime motivated behavior. In contrast to emotional expressions of anger which signal threat and where the organism may be tuned to automatically respond accordingly (Dimberg & Öhman, 1996).

The significant differences between facial expressions with face direction left also fits with both the model from Adams and Kleck (2005) and the self-relevance model (N’Diaye et al., 2009), as fearful facial expressions with left face direction elicited greater startle than happy and angry facial expressions with the same face direction. However, the significant greater startle response to neutral facial expression with left face direction compared with angry expressions with the same direction is harder to explain based on the self-relevance model. These results were not as expected. That is, averted neutral expression produced a similar rate of startle potentiation compared with averted fearful faces, and greater startle than averted anger. As far as we know, this is the first study investigating the startle reaction to faces with a neutral expression directed to the side. As such, we can only speculate to the reason behind this finding. However, Adams and Kleck (2005) found that an averted gaze increased the likeliness for a neutral facial expression to be attributed as a fearful expression. As such, it could be that a neutral facial expression within an averted face was decoded as an avoidance-motivated emotion, and, subsequently, produced similar startle responses as averted fear.

Gaze direction may also have been an important factor in the present study. Indeed, pictures of faces directed to the side indirectly produced an averted gaze in comparison to facial expressions with frontal face direction and forward gaze direction. As it is impossible to disentangle the effect of face direction and gaze direction in the present study, it remains a question for further research whether face or gaze direction is the crucial factor in this regard.

The results for the frontal face direction at the late lead interval revealed greater startle to angry faces, compared with fearful and neutral. These results are similar to the results of Hess et al. (2007) and Springer et al. (2007) who also found potentiated startle to angry facial expressions. Moreover, these results are in line with Alpers et al. (2011), who found increased startle to angry facial expressions compared with neutral ones, but not compared with happy expressions. Similar to Springer et al. (2007), we found potentiated startle to angry faces but not fearful ones. In sum, the results of the present study are in line with previous findings for frontal directed faces and startle probes with standard lead interval.

The results also revealed a significant main effect of lead interval with greater startle to the long lead interval compared with the short lead interval. This is probably caused by prepulse inhibition (PPI; Graham, 1975) reducing the responses for the short lead interval. Bradley et al. (1993) showed that pictures can serve as prepulses and reported PPI to neutral, pleasant, and unpleasant pictures at a 300 ms lead interval. In the present study, follow-up analyses to the interaction of Face Direction by Emotion by Lead Interval revealed greater startle to a neutral facial expression than a fearful facial expression, when faces were directed forward. This is in line with the results from Bradley et al. (1993) and Bradley et al. (2006) who found less PPI, and hence larger startle responses, to neutral stimuli compared with pleasant and unpleasant stimuli.

The notion from Adams and Kleck (2005) that gaze direction signaling the same emotional motivation enhances the perception of emotion in the face could possibly explain the other results from the PPI data. That is, startle responses were larger to neutral facial expression when the face was directed forward compared with the same facial expression when the face was directed to the side. As mentioned earlier, it is possible that a neutral face directed to the side is decoded somewhat similar to a fearful face in the same direction. For face direction to the side, angry facial expression is possibly less emotional significant compared with fearful and neutral expression. However, the larger response to averted angry compared averted happy is not as fitting to the Adams and Kleck (2005) model. In sum, the results from the startle data for the short lead interval seems to add moderate support to the idea that emotional prepulses inhibits startle more than less emotional prepulses. However, as Duval et al. (2013) showed, other aspects not investigated in the present study, such as the sex of the expresser, could influence the results regarding picture of faces as prepulses.

In the valence data, the main effect of Emotion was significant, and contrast analyses revealed that the happy faces was rated as more positive than the neutral ones. Neutral was rated as more positive than fearful, which in turn was rated as more positive than angry. It is notable that angry faces was rated as most negative, and this could possibly explain why angry faces elicited more startle than fearful ones. However, as the startle data showed a much more complicated pattern of results, this is not likely. It is also worth noting that angry and fearful facial expressions with left face direction had a similar valence rating as their frontal-face-direction equivalents. Therefore, it is not likely that the larger startle to facial expressions with frontal face direction could be explained by valence.

Analysis of the arousal data revealed a main effect of emotion. Follow-up analyses revealed more arousal to angry and fearful facial expressions compared with neutral and happy. It is not common for happy facial expressions to be equal in arousal to neutral expressions. However, it is possible that the Norwegian words used in this study describing the end points on the VAS for arousal were not identical in meaning to the English counterparts, which may explain this atypical result. There is no good Norwegian word for arousal and the word used is more akin to “activation.”

As described in the previous two paragraphs, taking face direction into account, the effect of the emotional facial expressions on startle and the subjective evaluations of the same expressions diverged. That is, startle was greater to angry facial expressions with frontal directed face compared with angry facial expression with the face directed to the side. In the subjective data (valence, arousal and dominance), there was no such difference. The same was found for happy faces. For fearful faces, there was a trend toward greater startle when the faces were directed to the side. Such a trend was not apparent in the subjective data. A similar difference between startle and subjective evaluations of valence, arousal, and dominance was reported by Paulus et al. (2014). They found potentiated startle to angry male faces compared with angry female faces, but no difference in subjective evaluations between genders. In the present study, the explanation may be that the participants were unable to report differences in subjective data to the more complex stimuli combining emotional stimuli and face direction. The participants may not have been aware of the emotional difference between these stimuli, despite the difference in startle responding. It has been shown that startle responses have been modulated by negative emotional pictures even when the participants failed to label the valence (Reagh & Knight, 2013). Similarly, studies from our lab has shown potentiated startle to a tone conditioned stimulus following fear conditioning, but no difference in subjective evaluations to the tone following conditioning (Åsli & Flaten, 2008; Åsli, Kulvedrøsten, Solbakken, & Flaten, 2009).

To conclude, the present study showed that pictures of angry facial expressions directed forward elicited greater startle responses than pictures of neutral and fearful expressions. This is in line with previous research reporting greater startle to pictures of angry faces. However, in the present study, pictures of emotional faces were also presented at an angle from the side. Responses to these pictures were different, with greater startle to fearful faces compared with angry and happy. In sum, these results lend support to the notion that startle responses to emotional faces follow the same pattern as behavioral data from Adams and Kleck (2005). That is, a direct gaze enhances the perception of approach-oriented emotions, and a averted gaze enhances the perception of avoidance-oriented emotions. However, as the present study did not differentiate between face and gaze direction, these results should be followed up by further experiments designed to investigate these factors.
